# The first identification of nesfatin-1-expressing neurons in the human bed nucleus of the stria terminalis

**DOI:** 10.1007/s00702-019-01984-3

**Published:** 2019-02-15

**Authors:** Artur Pałasz, Katarzyna Bogus, Aleksandra Suszka-Świtek, Andrzej Kaśkosz, Shirley Saint-Remy, Aneta Piwowarczyk-Nowak, Łukasz Filipczyk, John J. Worthington, Kinga Mordecka-Chamera, Karol Kostro, Grzegorz Bajor, Ryszard Wiaderkiewicz

**Affiliations:** 1 Department of Histology, School of Medicine in Katowice, Medical University of Silesia, ul. Medyków 18, 40-752 Katowice, Poland; 2 Department of Anatomy, School of Medicine in Katowice, Medical University of Silesia, ul. Medyków 18, 40-752 Katowice, Poland; 3 American Medical Student Association (AMSA), School of Medicine in Katowice, Medical University of Silesia, ul. Medyków 18, 40-752 Katowice, Poland; 4 Division of Biomedical and Life Sciences, Faculty of Health and Medicine, Lancaster University, Lancaster, LA1 4YQ UK

**Keywords:** BNST, Nesfatin-1, Brain, Neuropeptides

## Abstract

Neuropeptides are involved in various brain activities being able to control a wide spectrum of higher mental functions. The purpose of this concise structural investigation was to detect the possible immunoreactivity of the novel multifunctional neuropeptide nesfatin-1 within the human bed nucleus of the stria terminalis (BNST). The BNST is involved in the mechanism of fear learning, integration of stress and reward circuits, and pathogenesis of addiction. Nesfatin-1-expressing neurons were identified for the first time in several regions of the BNST using both immunohistochemical and fluorescent methods. This may implicate a potential contribution of this neuropeptide to the BNST-related mechanisms of stress/reward responses in the human brain.

## Introduction

The bed nucleus of the stria terminalis (BNST), a part of the so-called “extended amygdala”, is a minor gray matter aggregation located in the medial basal forebrain of vertebrate species. Accumulating evidence prove that this intriguing structure plays a crucial role in the integration of stress and reward signaling, generation of anxiety responses, and regulation of fear learning (Harris et al. 2018; Pelrine et al. 2016; Rodriguez-Sierra et al. 2016). This maybe highly involved in the neuromechanism of addiction and feeding behavior (Ch’ng et al. 2018; Avery et al. 2016; Pleil et al. 2016). The potential role of BNST-related circuits in the origin of some psychopathic traits has also been postulated (Schiltz et al. 2007). Both the human and animal BNST are functionally connected with limbic structures, thalamic nuclei, and basal ganglia, while newly reported connections with temporal and paracingulate cortex are exclusive to the human brain (Avery et al. 2014). The human BNST, a relatively small (average volume ~ 180–190 mm^3^) droplet-shaped neural association is located in the central forebrain and subdivided along a medial–lateral axis consisting of four areas: medial (BNSTM), central (BNSTC), lateral (BNSTL), and ventral (BNSTV) (Theiss et al. 2017). Interestingly, the BNSTC represents a distinct sexual dimorphism, being larger in men than in women (Swaab 2007; Chung et al. 2002). The detailed neuropeptide profile of BNST has been previously reviewed (Kash et al. 2015). The BNSTM has a dense noradrenergic innervation (high β-hydroxylase immunoreactivity), the BNSTC contains an abundant population of somatostatin (SOM) neurons, whereas the more heterogenic BNSTL is characterized by SOM, cholecystokinin (CCK), NPY, and neurotensin expression (Walter et al. 1991; Martin et al. 1991). On the other hand, oxytocin signaling in the rat BNST seems to be involved in the mechanism of social recognition in rats and microinjections of oxytocin into these structures enhanced social memory in male, but not female animals (Dumais et al. 2016).

Nesfatin-1 is a recently discovered NEFA/nucleobindin-2 (NUCB2)-derived multifunctional neuropeptide (Schalla and Stengel 2018; Pałasz et al. 2012). Nesfatin-1 is known as a potent anorexigenic factor, inducing satiety, and inhibiting food and water intake (Wernecke et al. 2014; Stengel and Tache 2013). Intriguingly, a number of recent studies demonstrate that nesfatin-1 plays an important role in other autonomic and mental functions such as sleep–wake regulation (Vas et al. 2013; Jego et al. 2012), anxiety or stress-related responses (Pałasz et al. 2018; Emmerzaal and Kozicz 2013; Merali et al. 2008), and may also be involved in the pathogenesis of some psychiatric disorders (Weibert et al. 2018; Xu et al. 2018; Shimizu and Mori 2013; Gunay et al. 2012; Ari et al. 2011). The rat hypothalamus, arcuate, paraventricular, and supraoptic nuclei as well as in dorsomedial and lateral hypothalamus are characterized by distinct expression of nesfatin-1. In addition, perikarya of the piriform, insular and cingulate cortex, amygdala, BNST, lateral septum and zona incerta also demonstrate nesfatin-1 immunoreactivity (Goebel-Stengel and Wang 2013; Stengel and Tache 2010). Nesfatin-1 neurons were also identified in cerebellum (Purkyne cells) and numerous brainstem structures including solitary tract, raphe nuclei, gigantocellular reticular nucleus, lateral parabrachial nucleus, nucleus ambiguous, and central gray (nucleus O). Despite the accumulating animal studies on nesfatin-1 its presence and action are so far understudied in the human brain structures. The aim of the current histological study was to detect the presumptive existence of nesfatin-1-expressing neurons in the human BNST. A number of psychiatric disorders may potentially be connected with impaired neuropeptide-dependent regulation in BNST, hence this morphological study offers potential mechanistic understanding of neuropsychiatric disease.

## Materials and methods

**Fig. 1:**
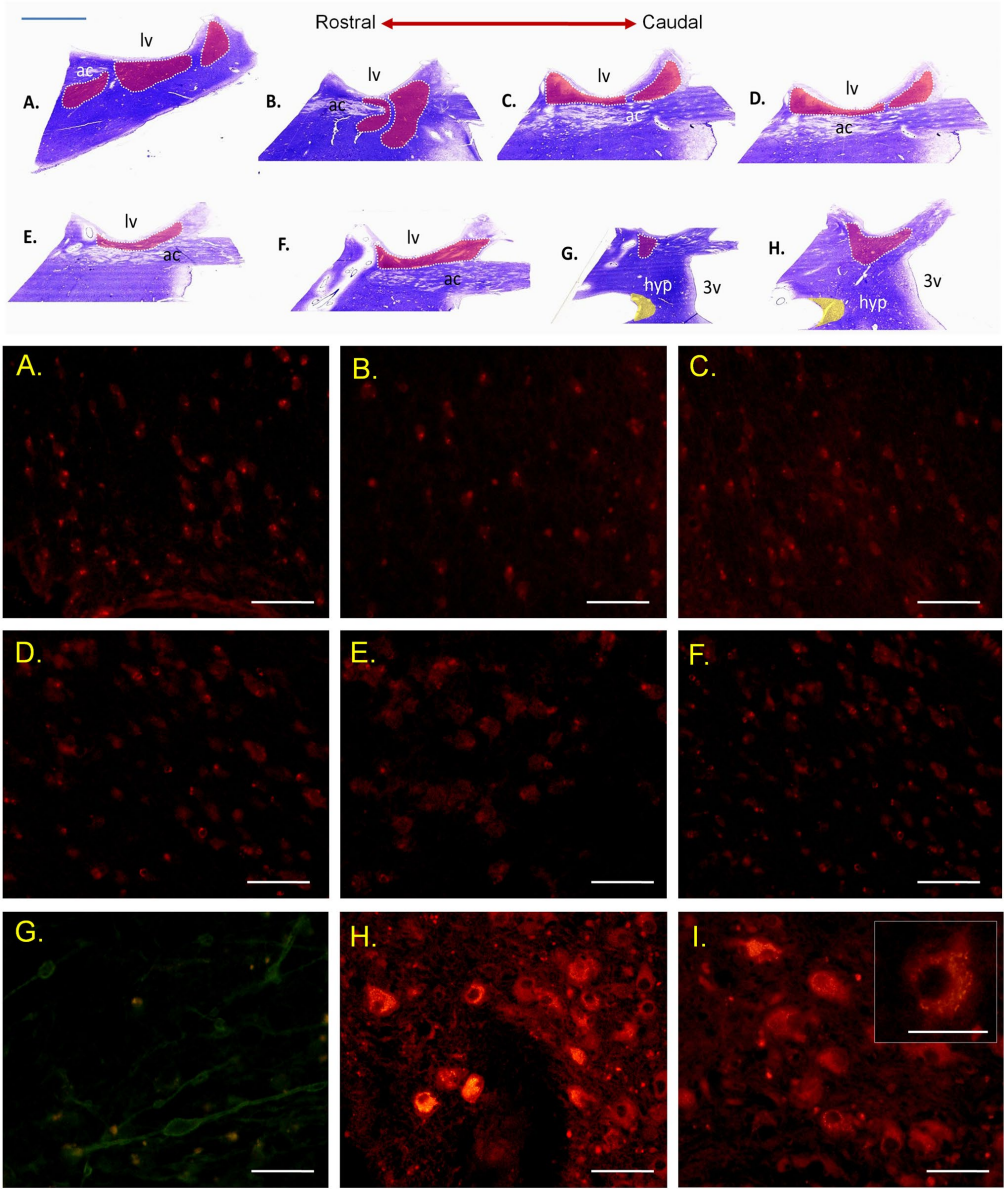
Overview of the analyzed coronal serial sections of the human brain pericommissural area at the level of BNST (red). Sections G and H contain the hypothalamic supraoptic nucleus (SON, yellow); anterior commissure, ac; lateral ventricle, lv; hypothalamus, hyp; third ventricle, 3v. Nissl staining. Scale bar: 5 mm. Immunofluorescence for nesfatin-1 in the human BNST. Cell bodies of the BNSTM (**a**–**c**) and BNSTC (**d**–**f**) subnuclei (TRITC). Neuropeptide immunopositive somata and fibers in the BNSTC (FITC,** g**). Magnocellular perikarya of the hypothalamic supraoptic nuclei (SON,** h**,** i**) with a distinct intensity of the fluorescent signal (a proven high level nesfatin-1 expression) represent an internal positive control. Scale bars: 200 µm and 100 µm (**i**, inset)

Studies were carried out on human brain tissue specimens with no neuropathological findings obtained within the Conscious Body Donation Program conducted by the Department of Anatomy at the Medical University of Silesia in Katowice. The brains were *postmortem* perfused and fixed with buffered solution 4% formaldehyde (pH 7.2–7.4) over a period of at least 3 months. The tissue samples containing BNST were precisely excised from two forebrain specimens according to Mai, Majtanik and Paxinos Atlas of the Human Brain (2015), dehydrated, embedded in paraffin, and finally sectioned on a microtome (Leica Microsystems, Germany) at 10-µm-thick serial slices (Fig. 1). The inferior BNST boundary was identified in the coronal plane by the superior side of the anterior commissure, superior was delineated by the most ventral edge of the caudate nucleus and lateral ventricle. Column of the fornix and internal capsule formed medial and lateral borderlines, respectively. The sections were deparaffinized with xylene and rehydrated to the 50% ethanol by successive changing of an alcohol gradient.

After rehydration, and subsequent antigen retrieval with citrate buffer (pH 4.0) solution (vector laboratories), sections were rinsed three times for 5 min in 0.05 M TBS-saline (pH 7.6) and placed in the 0.1% buffered Triton X-100 (Sigma) to improve the antibodies penetration and reaction quality by reducing surface tension of aqueous solutions during immunohistochemistry. They were blocked with 10% goat serum and incubated overnight at 4 °C with a mouse monoclonal antibody (I) against rat nesfatin-1 (1:1000, Enzo Life Sciences, IF) or rabbit anti-rat nesfatin-1 antibody II (1:5000 Phoenix Pharmaceuticals, IHC-P/IF). We decided to apply two antibodies both mono- and polyclonal comparatively to improve the immunostaining reliability. Cross-reactivity of the antibody II with the human cells was reported previously (Ramanjaneya et al. 2010). Sections were additionally treated with TrueBlack® Lipofuscin Autofluorescence Quencher (Biotium, Hayward, CA, USA) to minimize unwanted intense lipofuscin signal. After incubation with primary antibodies, brain sections were kept in darkness with appropriate secondary antibodies: goat anti-mouse and goat anti-rabbit labeled with TRITC or FITC (1:200, Abcam), respectively, and mounted on slides with DAPI-containing medium. Neurosecretory cells of the hypothalamic supraoptic nuclei (SON) with a distinct intensity of the fluorescent signal (a proven high level nesfatin-1 expression) represent an internal positive control. Alternatively, primary antibody I was followed by biotinylated rabbit anti-goat secondary antibody, and then an avidin-biotin-horseradish peroxidase complex (Vectastain ABC kit, Vector Labs). Finally, 3,3′-diaminobenzidine (DAB) was used to visualize the reaction. All sections were mounted on glass slides, dehydrated, and coverslipped. For basic neurostructural evaluation representative sections were stained with Nissl method in 1% of water solution of Cresyl violet for 60 min. After rinsing and differentiation by acetic acid and mounting with DPX, sections were preserved with cover glasses. All images were captured on a Nikon Coolpix fluorescent optic systems and processed using Image ProPlus software (Media Cybernetics, USA). Despite the much extended process of tissue fixation, the general neuronal morphology was sufficient to perform immunohistochemical analysis. The BNST cyto- and chemoarchitecture was analyzed and immunopositive cells were counted using ImageJ 1.43u software. The anatomical coordinates and structure of BNST were defined according to Mai human brain atlas (Mai et al. 2015). Positive cells were divided per area of the analyzed BNST subdivision; medial (BSTM), ventral (BSTV) and lateral (BSTL) to obtain the density of immune-positive cells per standardized area (0.2 mm^2^). Data are presented as a mean ± standard error of the mean (SEM).

## Results and discussion

**Fig. 2:**
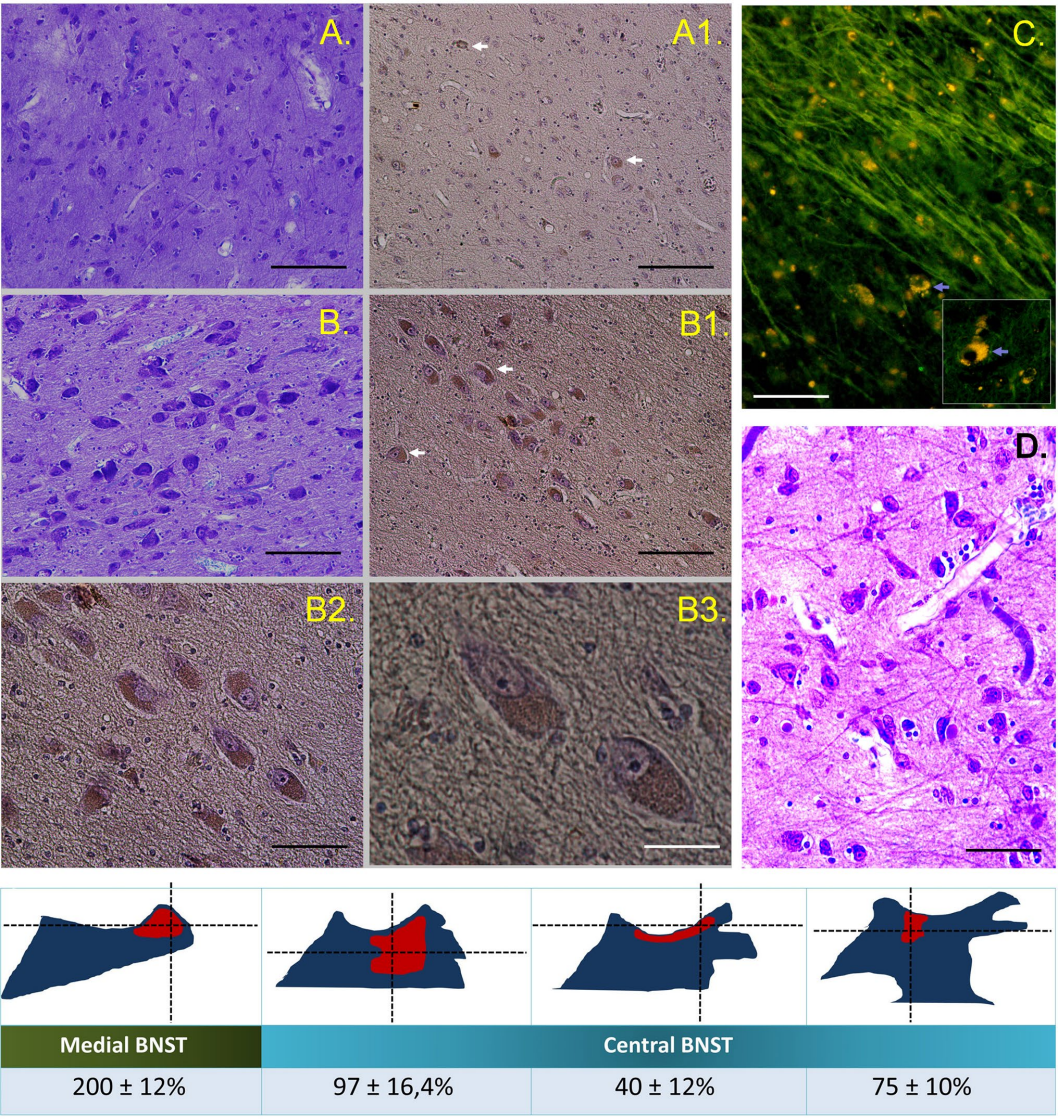
Immunohistochemical reaction for nesfatin-1 in the human BNST. General microstructure and cell diversity of the central (BNST, **c, a**) and ventral (BNSTV, **b, d**) subdivision; Nissl staining. Neuropeptide immunopositive perikarya in the respective regions (A1, A2, white arrows); DAB reaction. A selection of neurones from the picture B1 under higher magnification (B2, B3). Immunopositive fibers in the BNSTC (fluorescence, FITC, **c**), numerous cell bodies contain deposits of lipofuscin with a strong yellow autofluorescence (inset, blue arrows). Mean number of nesfatin-1 immunopositive cells per standardized frame (0.2 mm^2^) located at the intersections of dotted lines (a preliminary analysis). Values for two BNST subnuclei taken from selected brain sections (see Fig. 1). Scale bars: 200 µm (A, A1, B, B1, D), 100 µm (B2), 50 µm (B3)

We have demonstrated for the first time nesfatin-1 immunoreactive neurons in the human BNST which suggests that this novel neuropeptide may be involved in functions realized by this brain region. Numerous nesfatin-1-positive neurons are present in the human BNST and their assemblies show different patterns of distribution in selected BNST subnuclei. Highest density of medium-sized (20–30 µm), nesfatin-1-immunoreactive cells were observed in the BNSTM sections; mean number per standardized frame area was 200 ± 12%. In the BNSTC, a smaller proportion of multiform neuropeptide immunopositive cells was found depending on the section plane starting of more rostral orientation (97 ± 16.4%, 40 ± 2% and 75 ± 10%, respectively, Figs. 1, 2). Of note, the relatively large (40–50 µm), oval-shaped perikarya of the ventral BNSTC exhibits high nesfatin-1 immunoreactivity (Fig. 2; b1–b3). The chemoarchitecture of nesfatin-1 neurons in the BNST seems to be similar to the distribution of NPY (Adrian et al. 1983), PACAP (Palkovitz et al. 1995), and dynorphin-A neurons (Poulin et al. 2009).

At present, there is no consensus in the role of nesfatin-1 to the function of human brain neuronal pathways. One can merely suppose that its role in the BNST may be potentially analogous to that revealed in the rodent brain. For instance, acute restrain stress is one of the main factors activating nesfatinergic neurons in the rat hypothalamus and selected brainstem structures, e.g., Edinger–Westphal, *locus coeruleus*, and solitary tract nuclei (Stengel et al. 2010a; Goebel et al. 2009). Intracerebroventricular injection of nesfatin-1-elevated blood pressure (Yosten and Samson 2009), whereas extended intraperitoneal administration of nesfatin-1 also facilitated anxiety in male rats (Ge et al. 2015). Furthermore, animals exposed to acute, but not chronic stress show an increase in both the mRNA expression of NUCB2/nesfatin-1, and corticotropin-releasing factor (CRF) within the hypothalamus (Xu et al. 2015). This may be especially important, given the distinct population of stress-related CRF neurons, including some oxytocin-regulated cells, is located within rat and human lateral BNST (Janecek and Dabrowska 2018; Silberman et al. 2013; Morin et al. 1999). Intriguingly, nesfatin-1/NUCB2 mRNA expression in the human Edinger–Westphal nuclei was significantly increased in suicidal victims (with no diagnosed psychiatric disorders) among males, whereas among females, this content was lower, compared to controls (Bloem et al. 2012). The possible role of nesfatin-1 signaling in the pathogenesis of sex-related depressive-like and anxiety behavior in the context of BNST function should be, therefore, taken into account. Given the small number of brain samples available for this study, it is imperative that further studies expand these findings to complement our initial report.

Our results demonstrate for the first time the presence of nesfatin-1 in the human limbic structures which could implicate a potential contribution of this factor to the central mechanisms of stress/reward responses. Currently, a role for nesfatin-1 in human BNST physiology remains speculative, but undoubtedly further studies on this novel regulatory factor, e.g., its coexpression with other important BNST neuropeptides such as CRF, NPY, somatostatin and analysis of potential functional relationship between these regulatory factors, strongly merit attention. Moreover, the 3-D visualization of several novel multifunctional neuropeptides (nesfatin-1, phoenixin, spexin) spatial distribution in the human BNST, amygdaloid complex, other limbic structures including several basal ganglia and hypothalamus should be of primary focus.
